# An investigation of the morphological, thermal, mechanical, and barrier properties of an active packaging containing micro‐ and nano‐sized ZnO particles

**DOI:** 10.1002/fsn3.3665

**Published:** 2023-09-07

**Authors:** Raihan Syarifa, Yasaman Esmaeili, Shima Jafarzadeh, Farhad Garavand, Shahrom Mahmud, Fazilah Ariffin

**Affiliations:** ^1^ School of Industrial Technology Universiti Sains Malaysia Minden Malaysia; ^2^ Department of Food Science and Technology, Isfahan (Khorasgan) Branch Islamic Azad University Isfahan Iran; ^3^ School of Engineering Edith Cowan University Joondalup Western Australia Australia; ^4^ Department of Food Chemistry and Technology Teagasc Moorepark Food Research Centre Fermoy Ireland; ^5^ School of Physics Universiti Sains Malaysia Minden Malaysia

**Keywords:** active packaging, physicochemical properties, sago, zinc oxide

## Abstract

Biodegradable films are extremely important for food packaging applications since they minimize environmental effects. However, their application areas are limited due to insufficient characteristics required for particular applications. The objective of the present research was to improve the properties of sago‐based biodegradable films embedded with nano‐ and micro‐ZnO (zinc oxide). Nano and micro‐ZnO were incorporated in the films at different percentages (1%, 3%, and 5%) in that the films were formed using the solvent casting method. The physicochemical, barrier, thermal, optical, morphology, and mechanical properties of sago‐based films were investigated. Adding 5% of micro‐ and nano‐ZnO significantly improved film thickness (0.162 and 0.150 mm, respectively) and WVP (4.40 and 5.64 (kg/s)/(m.Pa), respectively) while the optical properties and thermal stability exhibited superior performance. Micro‐ZnO particles improved the mechanical properties of sago‐based biodegradable films with the tensile strength reaching 6.173 MPa. Moreover, sago‐based nano‐ZnO films showed excellent UV‐shielding performance and relatively good visible‐light transmittance. This study suggested that sago biodegradable film incorporated with micro‐ZnO could be an excellent alternative to petroleum‐based plastic packaging.

## Graphical abstract

The objective of this research is to improve sago‐based biodegradable film embedded with nano‐ and micro‐ZnO (zinc oxide), as well as estimate the properties of the films. Different percentages of nano‐ and micro‐ZnO (1%, 3%, and 5%) were incorporated, and films were formed using the solvent‐casting method.

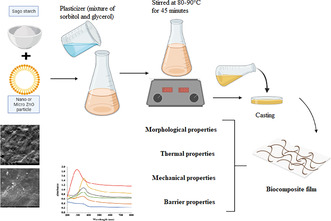

## 1 INTRODUCTION

Plastics are used most extensively in the packaging industry, with over 90% of flexible packaging made from plastics (Garavand, Jafarzadeh, et al., 2022; Garavand, Khodaei, et al., 2022). In recent years, the use of plastics in the food packaging industry has grown exponentially due to their low cost and good resistance against mechanical and environmental factors. Despite such desirable attributes, food packaging accounts for most global solid waste, with fewer than 12% recycled plastics and ~95% discarded after one‐time use (Samani et al., 2023). Plastic waste is one of the most common types of debris found in coastal environments, and every year a huge amount of plastics leak into marine areas (Ekramian et al., 2021; Garavand, Jafarzadeh, et al., 2022; Garavand, Khodaei, et al., 2022). Petroleum‐based plastic materials are particularly harmful because they take centuries to degrade (Ghalambor et al., 2022; Sahraee et al., 2020; Syafiq et al., 2021). It is predicted that the amounts of greenhouse gases produced as a consequence of plastic production and incineration will jump from 850 million tons in 2021 to 2.8 billion tons in 2050, significantly contributing to climate change. The significant damages that plastics pose to the environment have drawn the attention of scholars and practitioners to plastic use reduction. The use of environmentally friendly materials for food packaging is an effective means to reduce the environmental problems posed by plastics. Researchers have been working on the development of eco‐friendly packaging using biodegradable materials, such as proteins and polysaccharides. Biodegradable packaging is the most effective solution for addressing the environmental issues imposed by plastics. Numerous biodegradable packages have been developed using natural materials such as polysaccharides (starch, chitosan, and cellulose), proteins (wheat gluten, whey proteins, soy proteins, and fish proteins), or a combination of both. For instance, Abdullah and Dong (2019) developed a biodegradable film using poly (vinyl) alcohol (PVA)/starch (ST). In another project, Haque and Naebe fabricated biodegradable packaging from cotton gin trash and polyvinyl alcohol (Abdullah & Dong, 2019). Among many natural sources of biodegradable packaging, starch has attracted the interest of the packaging industry due to its low cost, lack of odor, taste, and color, and excellent filming properties. Thus, we chose sago starch for this work. Sago starch has a relatively high amylose content, which makes it more mechanically stable than other starches. While sago starch has excellent properties comparable to petroleum‐based plastics, it has certain limitations, such as high permeability. Fillers can be incorporated into them to address their limitations and enhance their properties.

Nano‐ and microparticles used in the biopolymer matrix remarkably strengthen the film's physicochemical, mechanical, and optical properties. According to Nafchi et al. (2012), among the metal oxides, ZnO, TiO2, MgO, and CaO have received interest because of their unique properties (Nafchi et al., 2012). Zinc oxide exhibits compelling antimicrobial characteristics even at low concentrations, along with high stability against elevated temperatures and remarkable UV absorbance properties, making it an intriguing agent (Noshirvani et al., 2017; Salarbashi et al., 2021). Moreover, it is considered safe and approved by the US Food and Drug Administration (FDA). In terms of price, nano‐zinc oxide comes at a higher cost than micro‐zinc oxide. The present study aimed to evaluate and compare the influence of nano‐ and micro‐zinc oxide based on the physicochemical, mechanical, optical, and thermal properties of sago starch‐based nanocomposites.

## 2 MATERIALS AND METHODS

### 2.1 Materials

Sago starch was obtained from SIM Company Sdn. Bhd. (Penang, Malaysia). Nano‐ZnO (20 nm) and micro‐ZnO were obtained from the School of Industrial Technology, USM. Food‐grade glycerol and sorbitol were obtained from R & M Marketing. Magnesium nitrate was obtained from QRëC Company.

### 2.2 Film preparation

Sago flour (4 g) was dispersed in 80 mL of distilled water using magnetic stirring. Then, 1.6 g of plasticizer, a mixture of sorbitol and glycerol (3:1), was added. Filler amounts of 1%, 3%, and 5% of nano‐ and micro‐ZnO, respectively, were added (Abdorreza et al., 2011). The dispersions were mixed and stirred for 1 h. Next, the mixtures were heated at 80–90°C for 45 min followed by cooling to room temperature, and 90 g of the sample was then cast on Perspex plates (16 × 16 cm^2^). The resulting films were left to dry for 12 h at room temperature and then placed in an oven set at 45–50°C for 10 h. The dried films were detached from the plates and stored in a desiccator (23 ± 2°C, RH 50%–55%).

### 2.3 Thickness

The thicknesses of the films were measured in five randomly selected locations around each film using a digital micrometer (Model No 73005; Mitutoya).

### 2.4 Moisture content

In order to determine the moisture content of the biodegradable film, about 50 mg of the film was initially kept at room temperature (25°C, 58% RH) for 2 days. Following that, the biodegradable films were dried for 1 day at 105°C in order to obtain their equilibrium weight. Moisture content was calculated using equation (1) (Xie et al., 2020):

$$\text{Moisture content}=\frac{M_{i}–M_{f}\;}{M_{i}\;}\times 100\;\;\;\;\;\;\;\;\;\;\;\;\;\;\;\;\;\;\;Eq(1)$$

As shown in the equation, *M*_i_ and *M*_f_ represent the weights of the dried samples at their initial and final weights, respectively. For each sample, three replicates were tested.

### 2.5 Optical properties

A colorimeter (Minolta CM‐3500D, Minolta Co. Ltd., Osaka, Japan) was used to measure the films' colors. In this experiment, L* (luminosity), b* (yellow to blue), and a* (red to green) were determined. For each film, three points were selected (Li et al., 2021).

### 2.6 Light transmission and absorbance

A UV‐1650 spectrophotometer (Model PC; Shimadzu) was used to determine the ultraviolet (UV) and visible‐light barrier properties of sago films. A spectrophotometer test cell was used to test film strips. As a reference, an empty test cell was used.

### 2.7 Film morphology

Gold was used to vacuum coat the samples to prepare the conditioned biodegradable sample for scanning electron microscope (SEM). A Leo Supra 50 VP scanning electron microscope (Carl‐Ziess. SMT) was used to visualize the surface microstructure of biodegradable films. An Oxford INCA 400 energy‐dispersive X‐ray system was used with the electron microscope.

### 2.8 Mechanical properties

Following ASTM D882‐10 (ASTM, 2010), tensile strength (TS), elastic modulus (EM), and elongation break (EB) were evaluated based on a texture analyzer (TA‐XT2, Stable Micro Systems) as described by Mousavi et al. (2022) in details. Five replicates were tested for each sample.

### 2.9 Thermogravimetric analysis

As part of a research project, 15 mg of samples were scanned by a thermogravimetric analyzer (TGA‐1; Perkin Elmer) at a temperature range from 40 to 800°C under a nitrogen atmosphere at a heating rate of 10°C/min.

### 2.10 Water vapor permeability (WVP)

ASTM E96‐05 standard (ASTM, 2005) was used to measure the WVP of the films gravimetrically according to Valizadeh et al. (2019) and Mousavi et al. (2021). The films were sealed over a circular opening in a permeation cell containing 6 mL distilled water; then, the cell was placed in a desiccator with silica gel. Weight loss of the glass permeation cell was measured every 2 h for 12 h to determine the amount of water transported through the film.

Equation (2) was used to determine the measured WVP of the films.

$$\mathrm{WVP}=\frac{\text{WVTP}\times L}{∆P\times A}\;\;\;\;\;\;\;\;\;\;\;\;\;\;\;\;\;\;\;Eq(2)$$

The WVTR is the water vapor transmission rate (g/d), *L* is the average thickness of the film (m), Δ*P* is the partial water vapor pressure variance (Pa) across the two sides of the film, and *A* is the area of the cup (m^2^).

Each sample was tested three times.

### 2.11 Statistical analysis

An experiment with three different lots of film samples was conducted in triplicates (*N* = 3) using a completely randomized design (CRD). In this study, data were presented as means ± standard deviation, and significance was determined by the probability value of ≤.05. ANOVA was carried out, and Duncan's multiple‐range test was used for comparisons of means. SPSS version 22.0 was used for the statistical analysis.

## 3 RESULTS AND DISCUSSIONS

### 3.1 Thickness

**FIGURE 1:**
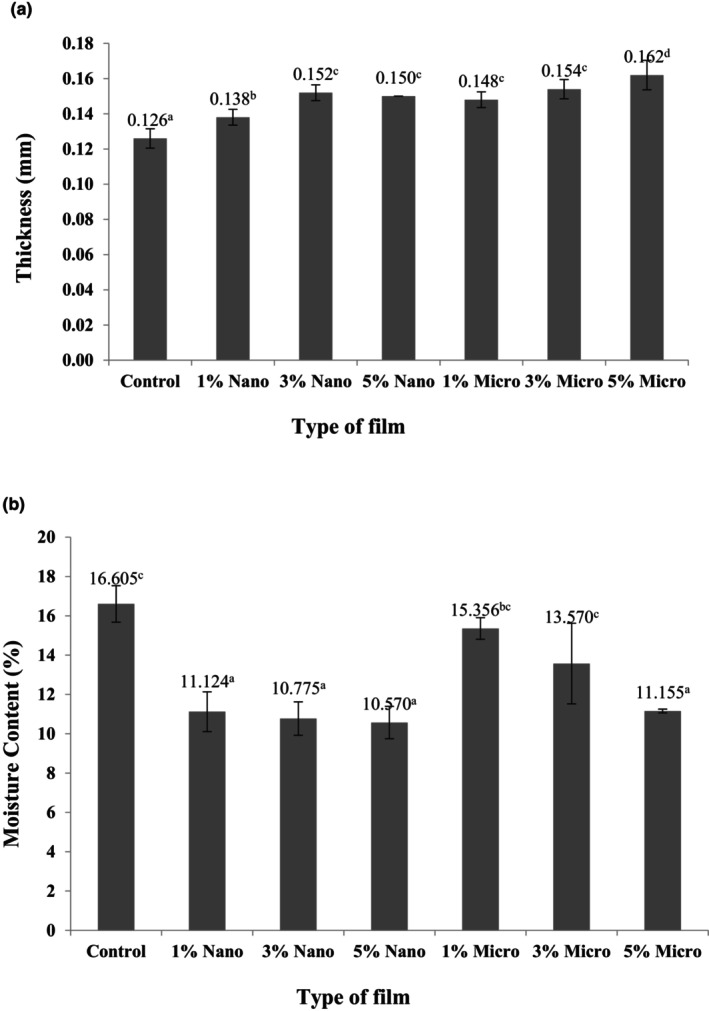
(a) Thickness of sago‐based biodegradable film and with nano‐/micro‐ZnO, (b) moisture content of sago‐based biodegradable film and with nano‐/micro‐ZnO. Mean (*N* = 5) and different letters within each column for each type are significantly different (*p* ≤ .05).

The thickness data of sago‐based biodegradable films embedded with different amounts of nano‐ and micro‐ZnO are shown in Figure 1a. The results showed that all seven types of films had significantly different film thickness values (*p* ≤ .05). The control film had the lowest mean compared to that of the other films. A relatively higher percentage of fillers could have led to an increase in the thickness mean that can be explained by a combined effect of zinc oxide and starch network, according to Jafarzadeh et al. (2019). In addition, Arfat et al. (2014) revealed that by increasing the amount of zinc oxide and *Ocimum basilicum* essential oil, the thickness of films (based on skin gelatin and fish isolates) was reported to increase (Arfat et al., 2014; Esmaeili et al., 2021). Kumar et al. synthesized ZnO nanoparticles (NPs) utilizing a green technique with *Mimusops elengi* fruit extract as a novel natural resource. The ZnO NPs were added into agar matrixes at 2% (w/w) and 4% (w/w) concentrations in that the ZnO NPs were employed to create bionanocomposite films in agar using solution‐casting technique. The ZnO NPs loading enhanced viscosity, which in turn raised film thickness. (Kumar et al., 2019).

In a different study, green‐generated ZnO NPs were added to hybrid nanocomposite films made of chitosan and gelatin. The control film thickness was ≈84.53 μm, and the addition of 2% ZnO NPs enhanced the thickness of the nanocomposite films (≈92.32 μm), which was known to occur in response to the higher viscosity of the nanocomposite solution (Kumar et al., 2020).

### 3.2 Moisture content

The moisture content of the films is an essential factor. The biodegradable film can last for a long time when the moisture content is low, while high moisture content can spoil the film due to favorable conditions for microbial growth. Based on Figure 1b, the moisture content is significantly different (*p* ≤ .05) among the seven film types. The highest percentage of moisture content was in the control film (16.605%). The results showed that the film's moisture content decreased as nano‐ and micro‐ZnO concentrations increased. The sago‐based biodegradable film with nano‐ZnO was shown to have less moisture content than the micro‐ZnO film. The reason for this is the interplay among plasticizer, biopolymer matrix, and nano‐ZnO, which diminished the hydroxyl group's accessibility to engage with water.

Arezoo et al. (2020) studied sago starch‐based film's functional properties by incorporating cinnamon essential oil (CEO) and nano‐titanium dioxide (TiO_2_‐N). They found that increasing either the CEO or TiO2‐N levels significantly decreased the solubility in water, moisture content, and water absorption capacity of the sago starch films (*p* ≤ .05). There was a decrease in moisture content from 12.96% to 8.04%, as well as a decrease in solubility from 25% to 13.7%. Nafchi et al. evaluated tapioca starch films' antimicrobial and functional properties using nano‐zinc oxide (ZnO‐N). According to the results, a reduction in moisture content was seen when different forms of ZnO NPs were incorporated into the film, and their concentrations were increased (*p* ≤ .05). Films containing ZnO nanospheres showed the highest reduction in moisture content (Tamimi et al., 2021).

### 3.3 Appearance (color)

Consumer acceptance can be affected by the film's appearance since color plays an important role, and consumers are more likely to accept a film with a good appearance. Three parameters were used, namely L*, a* and b*, for color measurement. According to Konica Minolta Sensing Americas (2017), Commission Internationale de I'Eclairage (CIE) stated that L*a*b* color space was modeled based on the theory that two colors cannot be red and green or yellow and blue at the same time. Thus, L* a* b* color space can be used to measure and compare the color intensities of objects in red, green, blue, or yellow. L* indicates the lightness of the sample, a* measures the difference in the red and green colors of the sample, and b* represents the yellower and bluer, respectively.

The parameters of color characteristics for sago‐based biodegradable film embedded with nano‐ and micro‐ZnO are shown in Table 1. Sago‐based biodegradable film (control) was transparent, odorless, and colorless. The color value for the control film was 93.707 ± 0.03 for lightness (L*), 0.083 ± 0.01 for redness (a*), and 6.147 ± 0.01 for yellowness (b*). The L*, a*, and b* have significant differences (*p* ≤ .05) among the sample for nano‐ and micro‐ZnO.

**TABLE 1:** Result for color analysis of different types of sago‐based biodegradable film.

Type of film	Color		
	L*	a*	b*
Control	93.707 ± 0.0388^f^	0.083 ± 0.015^a^	6.147 ± 0.012^b^
1% Nano‐ZnO	93.640 ± 0.040^f^	0.207 ± 0.015^d^	6.410 ± 0.100^a^
3% Nano‐ZnO	93.413 ± 0.012^e^	0.130 ± 0.010^b^	6.837 ± 0.012^f^
5% Nano‐ZnO	92.780 ± 0.020^d^	0.173 ± 0.006^c^	6.987 ± 0.015^g^
1% Micro‐ZnO	91.907 ± 0.021^c^	0.073 ± 0.021^a^	6.020 ± 0.017^c^
3% Micro‐ZnO	88.557 ± 0.104^b^	0.300 ± 0.010^e^	7.597 ± 0.023^e^
5% Micro‐ZnO	85.463 ± 0.025^a^	0.710 ± 0.010^f^	10.043 ± 0.012^d^

*Note*: Means ± standard deviation (*N* = 3) with different letter are significantly different (*p* ≤ .05). L* refers to lightness, a* refers to redness, while b* refers to yellowness.

L* value decreased, and the b* value increased by increasing the percentage of the fillers for both nano‐ and micro‐ZnO‐containing films. The a* value of films increased as the percentage of micro‐ZnO increased. In contrast, the a* value of films with nano‐ZnO decreased from 1% to 3% nano‐ZnO and slightly increased for 5% nano‐ZnO. This finding is in agreement with the research done by Jafarzadeh et al. (2016) in that the b* value increased and the L* value decreased with increasing filler content in the film. Sani et al. developed a nanocomposite film composed of chitosan, zinc oxide, and *Melissa officinalis* essential oil and they reported that as the concentration range of *Melissa* essential oil and ZnO NPs increased, the amount of L* decreased and a* increased, causing the color of the film to be yellowish (Sani et al., 2019). By incorporating sago starch films with TiO_2_‐N and cinnamon essential oil (CEO), Mizani et al. discovered that the lightness (L*) of the films decreased considerably with increasing TiO_2_‐N and CEO content, with the effects of TiO_2_‐N being more noticeable than those of CEO on the lightness of the sago starch films. Significant increases in yellowness (b*) were also found with the addition of CEO and TiO_2_‐N. The increase in yellowness was linked to CEO color (Arezoo et al., 2020). An investigation was conducted by Liu et al. on the effect of zinc oxide nanoparticles (ZnO NPs) and antioxidants of bamboo leaves (AOBs) toward chitosan films. ZnO slightly impacted the color parameters of chitosan films while the control film was transparent. As a result of the incorporation of AOB, there was a significant decrease (*p* < .05) in the lightness (L*) and redness (a*), but an increase in the yellowness (b*) of films was observed (Liu et al., 2021).

### 3.4 Thermogravimetric analysis

**FIGURE 2:**
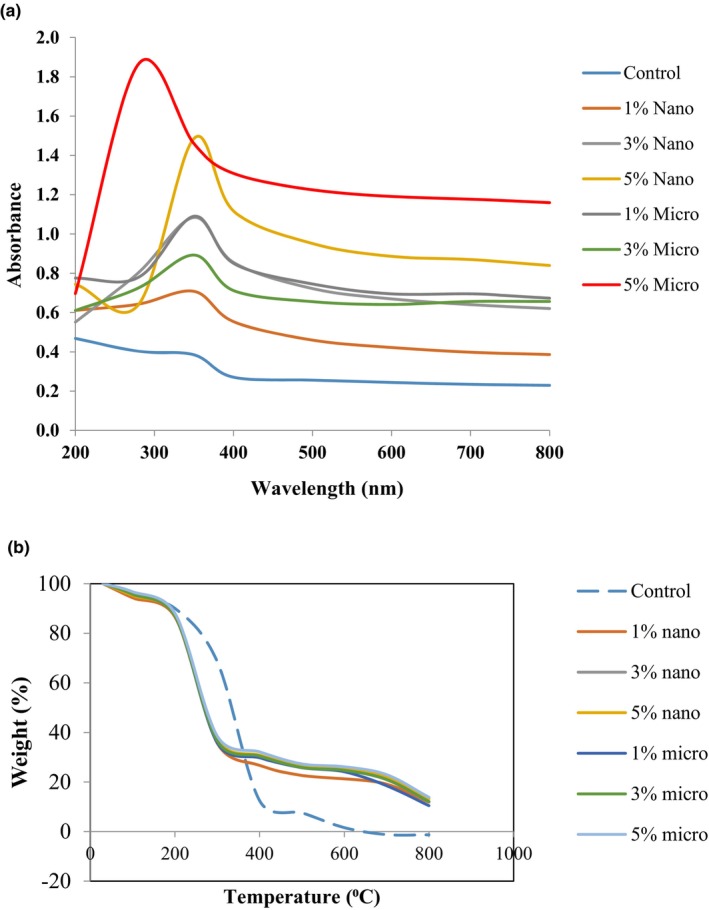
(a) UV–vis absorbance spectra of sago‐based biodegradable film and films embedded with various nano‐ and micro‐ZnO percentages. (b) Decomposition curves of sago‐based biodegradable films and films embedded with various nano‐ and micro‐ZnO percentages.

In terms of thermal analysis, thermogravimetry measures change in the physical and chemical properties of materials as a function of either increasing temperature (with a constant rate of growth) or over time (with continuous mass loss and constant temperature). Polymers maintain thermal stability until the initiation of the decomposition process. There are two types of thermal decomposition processes that are commonly acknowledged for polymers: chain polymerization and random decomposition. The liberation of monomer units from a chain end or a weak link, which essentially constitutes the inverse process of polymerization, is referred to as chain depolymerization. This process is commonly known as deportation or unzipping. Random degradation occurs when chains rupture at random points along the chains, resulting in a mixture of fragments. There is a possibility of these two processes occurring separately or in combination; the latter is a more common scenario. In both cases, sample mass losses can be measured using a thermogravimeter. Based on Figure 2b, the sample was completely burned without leftover residue in the crucible. All sago‐based biodegradable films embedded with nano‐/micro‐ZnO had higher thermal stability than that of the control film.

### 3.5 Light absorbance and transmission

In food packaging, light absorption is essential to preserve food quality by blocking out light. The light absorption and transmission of the sago‐based biodegradable film were determined by measuring the spectra of films using a UV–Vis spectrophotometer, and the result is shown in Figure 2a and Table 2. The UV region has been categorized into three zones as outlined below: UVC (100–280 nm), UVB (280–320 nm), and UVA (320–400 nm) (Jafarzadeh et al., 2017). The control film showed no absorption peaks between 200 and 350 nm. However, the films embedded with ZnO revealed a clear absorption peak. Most of the film showed a peak at UVA (320–400 nm), except for the control film, the intensity absorbance spectrum slightly shifted to the left, which is at the lower wavelength region (UVB). We can postulate that both sago‐based biodegradable films containing 5% micro‐ZnO and 5% nano‐ZnO may effectively block UV in packaged foods. Nanoscale‐ZnO particles showed superior UV‐blocking efficacy than that of larger particles ZnO (micro‐size) due to their high surface area‐to‐volume ratio and ability to scatter light. Overall, this study confirmed the suitability of ZnO for blocking UV in food packaging, particularly in oily food packaging.

**TABLE 2:** UV–vis transmittance spectra of sago‐based biodegradable film and films embedded with various nano‐ and micro‐ZnO percentages.

Samples	Wavelength							
	200	280	350	400	500	600	700	800
Control	31.5877	28.068	44.0471	54.1911	55.9733	57.487	60.083	60.7015
1% Nano	21.5535	24.9146	18.7419	28.6377	35.4411	36.6027	40.9383	42.1021
3% Nano	19.5028	11.2101	7.2771	14.0422	18.9941	21.4071	23.4497	24.5361
5% Nano	16.8457	8.8664	3.6255	7.255	10.6283	12.439	14.0625	15.0635
1% Micro	14.5874	16.6829	13.2528	13.3138	17.0166	19.2261	21.0002	22.0825
3% Micro	6.4046	14.6416	8.8135	18.7256	21.3826	22.1476	22.8271	22.8475
5% Micro	13.6108	13.9811	3.1372	4.7648	5.8797	6.425	6.8644	7.1004

In recent study, a film based on semi‐refined iota carrageenan/SiO_2_‐ZnO bionanocomposite with cassava starch addition was produced and applied to chicken meat packaging. The addition of NPs boosted UV protection in samples considerably (*p* < .05) (Praseptiangga et al., 2021). Additionally, Liu et al. observed a profound decrease in light transmission when ZnO and AOB were incorporated into chitosan films, mainly because UV–vis radiation was strongly absorbed by flavonoids. By increasing the concentration of AOB, the film became significantly more light resistant. As a result, chitosan/ZnO/AOB films had excellent potential for food packaging since they have excellent light barrier properties (Liu et al., 2021). A UV‐blocking packaging material was also produced by incorporating TiO_2_‐N into polystyrene, according to Altın & Sökmen (2014). Based on these findings, ZnO‐reinforced biopolymer films can be used as UV‐blocking films in food packaging

### 3.6 Mechanical properties

Tensile strength (TS), elongation at break (EB), and Young's modulus (YM) are the three main mechanical properties. TS refers to the maximum stress a material can handle, while EB refers to the percentage increase in length that a material will achieve before breaking. The YM measures the material's elasticity, or its ability to deform, and how much power it takes. These attributes are crucial for packaging material to prevent the packaged food from degradation due to mechanical damage and to preserve its integrity throughout the storage and logistic procedures (Oymaci & Altinkaya, 2016). The mechanical characteristics of films are intricately linked to the arrangement and concentration of the intra‐ and intermolecular connections among the polymer chains in the film matrix. Table 3 shows the mechanical properties of sago‐based biodegradable films containing nano‐ and micro‐ZnO. The TS of films containing nano‐ZnO decreased when the amount of nano‐ZnO was raised. In contrast, as micro‐ZnO was added to the films, the TS increased. Among all types of film, the EB has no significant difference (*p* > .05). The values of YM were increased with increasing micro‐ZnO content and samples with 5% micro‐ZnO illustrated the highest YM (183.658 MPa). However, by increasing nano‐ZnO content in the films, the YM in samples dramatically decreased (*p* ≤ .05).

**TABLE 3:** Mechanical and WVP properties of sago‐based composite films.

Type of film	TS (MPa)	EB (%)	YM (MPa)	WVP × 10^7^ (kg/s)/(m.Pa)
Control	5.447 ± 0.417^c^	181.095 ± 9.840^a^	170.540 ± 5.420^a^	7.61 ± 0.30^a^
1% Nano	6.078 ± 0.265^d^	183.218 ± 10.553^a^	179.215 ± 5.875b	6.81 ± 0.26^b^
3% Nano	5.222 ± 0.382^bc^	179.694 ± 3.463^a^	168.837 ± 5.215^c^	5.93 ± 0.26^c^
5% Nano	5.026 ± 0.1573^ab^	180.213 ± 6.430^a^	159.887 ± 5.699^cd^	5.64 ± 0.23^d^
1% Micro	4.866 ± 0.135^ab^	181.091 ± 6.604^a^	97.269 ± 5.540^b^	5.35 ± 0.23^c^
3% Micro	4.681 ± 0.205^a^	181.449 ± 4.603^a^	86.720 ± 5.065^d^	4.93 ± 0.25^e^
5% Micro	6.173 ± 0.256^d^	174.315 ± 4.273^a^	183.658 ± 5.678^cd^	4.40 ± 0.19^e^

*Note*: Means ± standard deviation (*N* = 5) with different letters are significantly different (*p* ≤ .05) and same letter is not significance different (*p* > .05).

Abbreviations: EB, elongation at break; TS, tensile strength; WVP, water vapor permeability; YM, young's modulus.

By increasing micro ZnO from 1% to 5%, the TS (6.173 MPa) and YM (183.658 MPa) of the films significantly increased compared to that of the control films. This result indicated that films containing micro‐ZnO had greater rigidity than that of the control film and films containing nano‐ZnO. Nandiyanto et al. investigated the effects of turmeric microparticles on corn‐starch‐based bioplastic material's mechanical and biodegradation properties. As a result, the measured Young's modulus decreased with increasing micro‐turmeric content (Nandiyanto et al., 2021).

Another research by Mizani et al. and previous studies on bio‐based films including various NPs such as SiO2, ZnO, and TiO_2_ revealed considerable increases in YM and TS and significant reductions in EB (Arezoo et al., 2020; Nafchi & Karim, 2013; Tabatabaei et al., 2018). Incorporating Ag NPs or clay into gelatin films can affect their mechanical properties, according to Kanmani et al. The TS of the gelatin/clay film and the gelatin/AgNPs/clay film increased significantly, whereas the EB of films decreased (Kanmani & Rhim, 2014). In contrast, according to Jafarzadeh and colleagues, the incorporation of 5% ZnO‐nr into semolina biopolymer films resulted in a notable improvement in both the tensile strength (5.13 MPa) and Young's modulus (143.51 MPa) of the bionanocomposite films, with an increase in the ZnO‐nr content from 1% to 5% (Jafarzadeh et al., 2017).

### 3.7 Water vapor permeability (WVP)

The water vapor transfer in films is determined by two key factors: solubility and permeability of water molecules. Water vapor permeability (WVP) is a crucial parameter for biodegradable films that significantly impact food degradation reactions.

The WVP of the films were determined and the results are shown in Table 3. A gradual decrease in WVP was observed in composite films as the ZnO content was increased. Sago‐based biodegradable film with nano‐ZnO had significant differences (*p* ≤ .05) compared to that of the control film and there was no significant difference (*p* > .05) between the film with 3% and 5% of micro‐ ZnO. The WVP for the control sago‐based biodegradable film was 7.61 × 10^7^, which decreased with the increase in the percentage of ZnO content. The sago‐based biodegradable film with 5% micro‐ZnO (4.40 × 107) showed the lowest WVP among all the films, possibly due to its relatively higher thickness than other films. In the same way, the addition of 1–3% (w/w) SiO_2_ to SPI‐based films resulted in a gradual decrease in WVP (Xu et al., 2019). According to Arfat et al. (2014), ZnO nanoparticles as well as basil leaf essential oils decreased the permeability of gelatin/isolated proteins to water vapor (Arfat et al., 2014). The results of this study are in agreement with those of Wang's team, who previously showed that hydrogen bonding between biopolymers decreases the WVP of quaternized chitosan and carboxymethyl cellulose films (Hu et al., 2016).

### 3.8 Film morphology

**FIGURE 3:**
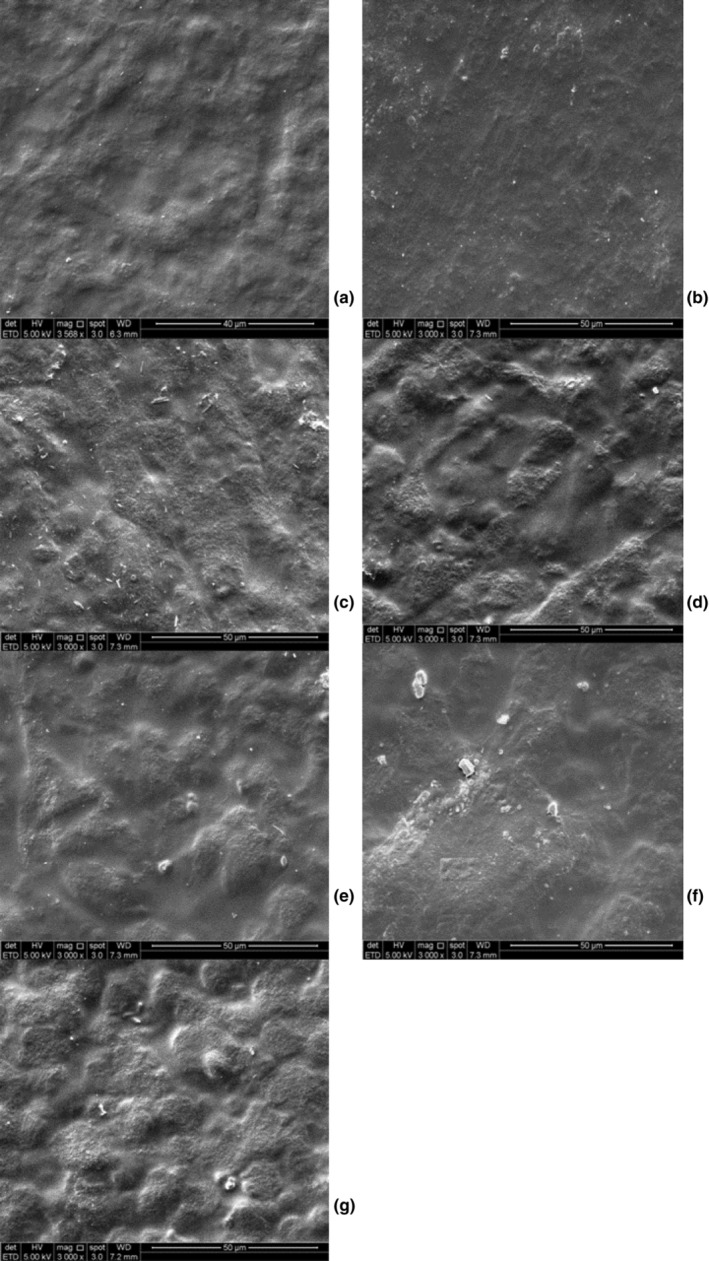
SEM micrograph of the surface of sago‐based biodegradable film. (a) Control film, (b) 1% nano‐ZnO, (c) 3% nano‐ZnO, (d) 5% nano‐ZnO, (e) 1% micro‐ZnO, (f) 3% micro‐ZnO, and (g) 5% micro‐ZnO.

The SEM micrograph of the surface of the neat sago film and the prepared biodegradable films are shown in Figure 3. There was less roughness in the surface structure of the control film than the ones in other films because there was no filler in the control film. The micrographs for film embedded with nano ZnO are smoother than the film embedded with micro‐ZnO. This was due to the different sizes of particles used whereby the finer particles can be easily dispersed in water compared to the larger ones.

## 4 CONCLUSION

Sago‐based biodegradable films embedded with nano‐ and micro‐ZnO were prepared at different ZnO percentages. Micro‐ and nano‐ZnO played an essential role in enhancing the physical properties of sago‐based biocomposites. Thickness, optical properties, thermal stability, and WVP were significantly improved by 5% of nano‐ and micro‐ZnO. SEM results clearly demonstrated that the films with nano‐ZnO appear homogenous and provide a smooth structure as the nano‐ZnO were uniformly dispersed in the film matrix. The sago‐based biodegradable films embedded with nano‐ZnO had higher absorbance in UVA (320 nm to 400 nm) regions than those with micro‐ZnO. WVP values in 3 and 5% micro‐ZnO samples were significantly lower than that of other samples (4.93 and 4.40 (kg/s)/(mPa), respectively). Mechanical properties of the sago‐based biodegradable film with 5% micro‐ZnO showed higher TS (6.173 Mpa), greater rigidity, and higher thickness (0.162 mm) than that of the control film and those with nano‐ZnO. To sum up, this study showed that micromaterials improved the sago‐based film's mechanical, barrier, thermal, and physicochemical properties. Moreover, micromaterials are easier to handle and process, more cost‐effective, more stable, and less prone to aggregation than nanomaterials.

## AUTHOR CONTRIBUTIONS

**Raihan Syarifa:** Investigation (equal); resources (equal); software (equal); writing – original draft (equal). **Yasaman Esmaeili:** Investigation (equal); methodology (equal); writing – review and editing (equal). **Shima Jafarzadeh:** Conceptualization (equal); project administration (equal); resources (equal); supervision (equal). **Farhad Garavand:** Supervision (equal); validation (equal); writing – review and editing (equal). **Shahrom Mahmud:** Methodology (equal); writing – review and editing (equal). **Fazilah Ariffin:** Project administration (equal); supervision (equal); writing – review and editing (equal).

## FUNDING INFORMATION

This research received no external funding.

## CONFLICT OF INTEREST STATEMENT

The authors declare no conflicts of interest.

## ETHICS STATEMENT

There was no need for ethics approval for the present research.

## Data Availability

The data that support the findings of this study are available on request from the corresponding author. The data are not publicly available due to privacy or ethical restrictions.
